# Cost-effectiveness of multigene sequencing test and treatment for metastatic non-small cell lung cancer: A unique setting in the initial adoption phase in Japan allowing testing only after standard treatment

**DOI:** 10.1016/j.heliyon.2024.e37867

**Published:** 2024-09-19

**Authors:** Naoko Shiraiwa, Shingo Kano

**Affiliations:** Graduate School of Frontier Sciences, The University of Tokyo, Department of Medical Informatics and Biosciences, Bio-innovation Policy, Japan

**Keywords:** Health technology assessment (HTA), Cost-effectiveness analysis, Next-generation sequencing (NGS), Non-small cell lung cancer (NSCLC), Oncology

## Abstract

**Objective:**

To clarify the cost-effectiveness of comprehensive diagnosis and treatment of metastatic non-small cell lung cancer in Japan, from initial diagnosis to post-standard treatment, using three different strategies.

**Methods:**

A decision tree was created using three diagnostic and treatment strategies, assuming that Foundation One CDx (F1CDx), a comprehensive genome panel, was introduced in Japan in June 2019. This comprehensive decision tree includes Markov models, cost-effectiveness analyses (CEA), and cost-utility analyses (CUA) of the three strategies from the perspective of Japanese payers. Specifically, Strategy1 involves single-gene testing at the initial diagnosis and F1CDx after standard treatment; Strategy2 involves only single-gene testing at the initial diagnosis; Strategy3 involves F1CDx at the initial diagnosis. The incremental cost-effectiveness ratios (ICERs) of the three strategies are estimated. Sensitivity analyses were performed to assess the uncertainty of the parameter settings.

**Results:**

Strategy3 was dominated for both CUA and CEA. The ICER/quality-adjusted life year (QALY) for Strategy2 versus Strategy1 was USD 13,734 (JPY 2,080,923, USD 1 = JPY 151.39 on April 1st, 2024), which is less than the willingness to pay of USD 45,900 (JPY 7,500,000), and Strategy2 was more cost-effective than Strategy1. F1CDx was not cost-effective compared to multiple simultaneous single tests at the initial diagnosis, either after standard treatment or at the initial diagnosis. Sensitivity analysis also showed that the most influential factor on the ICER for both CEA and CUA was treatment cost.

**Conclusions:**

From both patient benefit and health economic perspectives, introducing F1CDx after standard treatment in June 2019 was not as cost-effective as multiple simultaneous single tests at the initial diagnosis but was more realistic from a health economic perspective than introducing F1CDx at the time of initial diagnosis. Therefore, the policy at the time of F1CDx introduction in Japan was appropriate from a short-term health-economic perspective.

## 1 Introduction

### 1.1 Circumstances surrounding cancer treatment and next generation sequencing test

Remarkable progress has been made in cancer treatments in recent years. For the treatment of metastatic non-small cell lung cancer (mNSCLC), many “driver genes” related to cancer onset and proliferation have been identified, and treatments tailored to each gene alteration have been developed. The proportion of patients with non-small cell lung cancer (NSCLC) harboring aberrations in driver oncogenes, such as *EGFR*, is 20–25 % in Europe and the United States, and approximately 50 % in Asia. In contrast, the proportions of patients harboring mutations in *ALK* and *ROS1* are approximately 5 % and 3 %, respectively, with no ethnic differences [1]. If a driver gene mutation is identified, a targeted gene mutation-tailored therapy is administered. Compared to chemotherapy, targeted treatments have been reported to extend overall survival (OS) [2].

As of 2023, two types of genetic tests are available to detect these genetic mutations: tests that detect a single genetic alteration, and those that simultaneously examine multiple genetic alterations. The expectation for a test that can identify multiple genetic alterations is that all genetic alterations can be examined only in the initial diagnostic specimen [3]. Tests examining multiple genetic alterations include next-generation sequencing (NGS) and reverse transcription-polymerase chain reaction (RT-PCR), which cover multiple driver genes. Various guidelines recommend NGS, both globally and in Japan. As of January 2023, insurance reimbursements in Japan covered FoundationOne Companion Diagnostic (F1CDx), FoundationOne Liquid (F1CDx Liquid), and Oncomine Dx Target Test (ODxTT) [3,4]. As of June 2019, when F1CDx and ODxTT were launched, F1CDx had a companion diagnostic (CDx) function across cancer types, including NSCLC, which is a test for the use of approved drugs under insurance and a comprehensive genome profile (CGP) test function covering 324 genes, whereas ODxTT had only a CDx function for *EGFR, ALK, ROS1,* and *BRAF* [4]. In Japan, CGP testing is conducted after the standard treatment or at the expected stage [4]. Under the Japanese national health insurance system, patients pay a certain percentage of the price, and the remainder is paid by the government [5]. As of 2019, the calculation of reimbursements has become more complex when the CDx function of F1CDx is used. The reimbursement plan assigned a total of JPY 560,000 to F1CDx. JPY 80,000 was approved when the specimen was submitted to the testing laboratory, with JPY 480,000 potentially approved after explaining the results to the patient, which occurred after the expert panel discussed the results [4]. As 4–6 weeks were required to return the results, the cost of the analysis was not reimbursed if the patient's condition deteriorated to a point where further treatment could not be considered. In this case, the associated hospital has to cover the costs [4]. Therefore, in clinical practice, F1CDx is used only after standard treatment and not for NGS testing. Approximately 10 % of patients with solid tumors undergo NGS testing after standard treatment and receive further treatment [6,7]. Subsequently, from April 2022, the CGP test fee was reduced to JPY 440,000 and that of the CGP evaluation and provision to JPY 120,000 [8]. In Japan, the setting that limits NGS testing, ideally used at initial diagnosis, to only after standard treatments, was implemented considering two constraints: the maintenance of universal health coverage and the capacity of expert panels [9,10]. However, the results of the health-economic evaluation of this choice are not publicly available. In this study, we conducted a health technology assessment (HTA) to evaluate whether the decision to introduce these policies was quantitatively correct from a cost-effectiveness perspective.

### 1.2 Status of HTA for NGS test and cancer treatment in Japan and other countries

In Japan, new drugs and medical devices approved by the Pharmaceutical and Medical Devices Agency are reimbursed by public health insurance without cost-effectiveness data [11]. Owing to the rapid increase in medical costs, the introduction of a cost-effectiveness evaluation system for pricing was discussed in 2012, and the system was officially introduced in April 2019 after a pilot program was conducted from 2016 to 2018 [12]. The application scope of the cost-effectiveness evaluation scheme in Japan is to determine the adjustment of drug prices and not the initial drug price[[11], [12], [13]]. Regarding the concept of healthcare costs in other countries, such as the United Kingdom, Australia, and Canada, the national agency that conducts the health technology assessment (HTA) evaluates both the effectiveness and cost-effectiveness of each drug and medical device and decides whether to approve the drug or medical device price [12,13]. As there is no governmental HTA agency in the United States, the Institute for Clinical and Economic Review, established in 2006, is responsible for evaluating new treatments and making recommendations [14].

Although some HTAs have been reported with the introduction of NGS in other countries, there are no reports of total verification from the time of initial diagnosis to the end of standard treatment [15,16]. However, in Japan, details of cost-effective simulations for NGS testing have not been made public.

Two HTA studies have reported the integration of diagnosis and treatment, including NGS, for cancers other than mNSCLC. First, a study that evaluated NGS versus single-gene testing at the initial diagnosis of melanoma in the United States found that NGS resulted in lower healthcare costs and a better quality of life for patients. Next, the cost of NGS was shown to have little effect on incremental costs. However, this study considered only first-line treatment, and not treatment after disease progression [17]. The second study evaluated the efficacy of HTA in patients with platinum-refractory ovarian cancer in the United States using NGS versus chemotherapy without NGS. The incremental cost-effectiveness ratio (ICER) per quality-adjusted life-year (QALY) with the NGS test versus without the NGS test was USD 479,303, which is above the maximum willingness to pay (WTP) of USD 10,000. The most influential factor was the cost of the targeted treatments. However, as this study did not use a Markov model and made many assumptions, such as utilities and transition probabilities, certainty was not considered robust [18].

Researchers in the United States have evaluated the cost-effectiveness of F1CDx compared to single-gene testing, including subsequent treatment options for approximately 6000 patients with mNSCLC, using a large database [15]. The cost-effectiveness of F1CDx for single genetic testing at the first diagnosis was moderate (USD 147,000 per life-year [LY]), with the United States threshold ranging from USD 50,000 to USD 200,000 per LY. However, this study used OS data from the initial treatment when calculating the expected LY, which may not accurately account for the effects of each treatment from the initial treatment until the end of the standard treatment. In addition, it was evaluated only for LYs, and not for QALYs. In Australia, LY and the ICER for lung cancer patients were calculated by comparing the case of ODxTT performed before the fourth-line treatment and cases in which chemotherapy or best supportive care (BSC) was administered without testing [16]. This study presented the HTA of the total diagnosis and treatment results when ODxTT was performed after standard treatment in Australia but did not cover the total treatment process from initial diagnosis to post-standard treatments.

In summary, existing studies on HTA using NGS did not answer the question, “When should an NGS test be performed?”, which arose when the genetic testing method shifted from single-gene testing to NGS. Owing to the difficulty in referencing utilities for each targeted therapy, some studies have evaluated only LYs and not QALYs. However, QALYs are essential for comparison with previous studies and for examining the patients’ quality of life.

The purpose of the current study was to conduct a total cost-effectiveness analysis (CEA) and cost-utility analysis (CUA) from initial diagnosis to post-standard treatment for mNSCLC for three possible options in Japan that varied according to the timing of genetic testing, including NGS testing, and to evaluate these options quantitatively using both LYs and QALYs.

## 2 Methods

### 2.1 Diagnostic and treatment strategies

From the perspective of healthcare payers, a decision-analytic model was developed to estimate the expected direct medical costs, LYs, and QALYs over a ten-year period when (1) single-gene testing was conducted at initial diagnosis and F1CDx was conducted after standard treatment, (2) single-gene testing was conducted at initial diagnosis, and (3) F1CDx was conducted at initial diagnosis. The diagnostic and treatment strategies used were as follows:

Strategy1 Single-gene testing was conducted at initial diagnosis, and F1CDx was conducted after standard treatment.

Strategy2 Single-gene testing conducted at initial diagnosis.

Strategy3 F1CDx conducted at initial diagnosis.

Based on the 2018 Guidelines for Diagnosis and Treatment of Lung Cancer [19], this study included patients with a performance status (PS) of 0–2 and <75 years. Standard treatment was defined as up to second-line treatment. Treatment in each line is the most recommended in the Guidelines for Diagnosis and Treatment of Lung Cancer. The treatment sequence for patients with EGFR mutations was osimertinib as 1st line and Cisplatin (CDDP)/pemetrexed as 2nd line. For patients with ALK mutations, alectinib is used as 1st line treatment and lorlatinib as 2nd line treatment. For both EGFR- and ALK-negative patients, carboplatin (CBDCA)/pemetrexed/pembrolizumab was used as 1st line treatment, and CDDP/pemetrexed as 2nd line treatment. Regarding Strategy1, off-label or investigational therapy is used as 3rd line treatment after standard treatment for patients who are actionable, according to the results of F1CDx. To simplify the process, when F1CDx was administered after standard treatment, we assumed that *NTRK* was positive and that the *NTRK* inhibitor entrectinib was administered. Another 3rd line treatment after the standard treatment for patients who are not actionable, according to the results of F1CDx, is docetaxel (DTX)/ramucirumab.

In Japan, at the time of the June 2019 launch, the indications for the CDx function of F1CDx in mNSCLC were *EGFR* and *ALK*; therefore, only *EGFR* and *ALK* were included in this study [20].

Parameters known at the end of December 2023 were used in the analysis (Table S1). A Markov model from a previous study [21] was used as a reference.

### 2.2 Model-based cost-effectiveness analysis and curve fitting

**Fig. 1:**
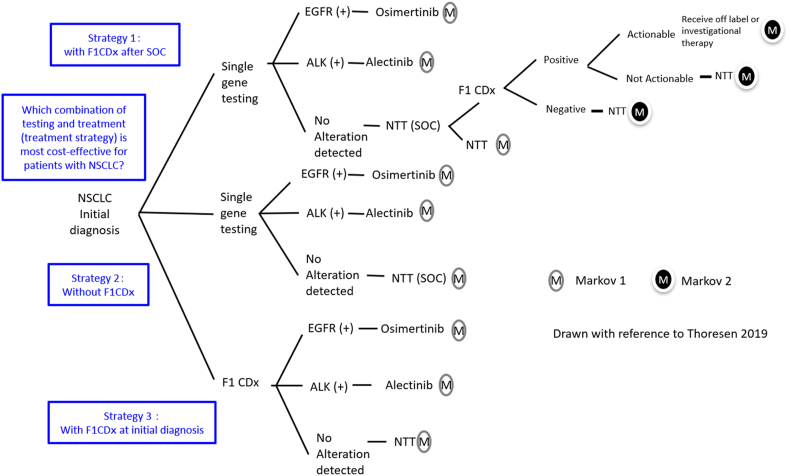
Decision Tree Strategy1: Simultaneous multi-single genetic tests conducted at the initial diagnosis and F1CDx after standard treatment. Strategy2: Simultaneous multi-single genetic tests conducted at initial diagnosis Strategy3: F1CDx conducted at initial diagnosis Non-small cell lung cancer (NSCLC), standard of care (SOC), non-targeted treatment (NTT).

**Fig. 2:**
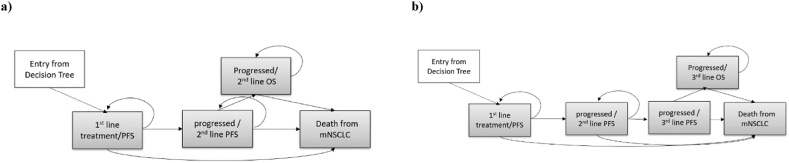
a) Markov Model 1, b) Markov Model 2 progression free survival (PFS); overall survival (OS).

A decision tree, including a Markov model, was created, CEA was performed under the above conditions, ICER was calculated, and sensitivity analysis was performed (Fig. 1, Fig. 2). The Markov model was adapted from Thoresen's model to accurately reflect the effects of first- and second-line treatments [21]. The outcome of CEA was LY and that of CUA was QALY. Progression-free survival (PFS) and OS were used as parameters to calculate LYs and QALYs. The mean cost-effectiveness/utility ratios were also calculated to identify whether each strategy alone costs more per survival year or per quality-adjusted survival year. The analysis was conducted using TreeAge Pro Healthcare (TreeAge Software, LLC, Williamstown, MA, USA). The length of each Markov model cycle was one month. The time horizon is 10 years. Markov model 1 was included in 2nd line treatment, and Markov model 2 was included for 3rd line treatment. To calculate the transition probability of each Markov model, curve fitting was performed using the Kaplan–Meier (KM) curve of PFS and OS for each treatment. Specifically, WebStabilizer (Ankit Rohatg, Pacifica, CA, USA) was used to extract information such as time and cumulative survival probability from the image data of each KM curve. Subsequently, a patient-level time-to-event dataset was created and the Weibull distribution was selected as the parametric function for curve fitting [22]. The Weibull distribution was selected because it was better suited for visual evaluation. Other possible functions, such as lognormal and logistics functions, were also executed; however, because there was no difference in the conclusion, the description was omitted. The parameters of the Weibull function obtained by curve fitting were identified, each KM curve was analyzed using the split survival (PartSA) model in TreeAge Pro Healthcare, and each transition probability was calculated by performing calibration. Calibration searches for the set of Markov model parameters that best matches the PartSA model output. The values of Scale and Shape, two parameters of the Weibull function calculated from the curve fitting of PFS and OS for each treatment at TreeAge Pro Healthcare, were introduced into the calibration model. The target data for PFS and OS (four points each in this study; at 5, 10, 15, and 20 months) were also introduced into the calibration model. Target data refers to the percentage of patients in the PFS and Dead states in the PFS and OS KM curves for each treatment. The calibration process was then performed to calculate the set of parameters of the Markov model, including the transition probabilities. The obtained parameter sets for the Markov models were introduced into the Markov models in the decision tree using TreeAge Pro Healthcare to calculate the CEA, CUA, and ICER. The calculation of ICER is described in Section 2.3 Economic Assumption.

### 2.3 Economic assumptions

Both CEA and CUA were also performed. Costs and outcomes were discounted at 2 % annually based on the Ministry of Health, Labor, and Welfare (MHLW) analysis guidelines [23]. Health outcomes are represented by LYs and QALYs. Increased cost-effectiveness or cost-utility was calculated by ranking the strategies in order of effectiveness or utility. Lower efficacy and equivalent or more expensive treatment strategies were considered “dominated” and were excluded from further analyses. The ICER was calculated for each treatment and compared to the next-best option. For each strategy, we calculated the ICER by dividing the difference in cost between the strategies by the difference in health status for comparison with the next-best strategy. The WTP was set at USD 49,500 (JPY 7,500,000)/QALY [24].

### 2.4 Model parameters

The annual treatment cost was calculated based on the premise of administration at the recommended dose from the Kyoto Encyclopedia of Genes and Genomes (KEGG) Medicus database (https://www.kegg.jp/kegg/medicus.html) and the package inserts of each drug. The costs of biopsies and genetic tests were calculated based on recommendations from the Medical Fee Database (http://shinryohoshu.mhlw.go.jp/shinryohoshu/).

Specifically, when calculating the doses of the intravenous drugs, we used the DuBois formula, assuming a male weighing 170 cm and 65 kg, and the JCOG body surface area calculation site (https://jcog.jp/doctor/tool/calc/). Annual drug costs are presented in Table S1. The costs of other laboratory tests, hospitalizations, radiography, CT scans, and treatment for adverse effects were omitted. In Strategy1, in which F1CDx was performed after standard treatment, all patients underwent a re-biopsy before F1CDx was performed. The clinical parameters were obtained from pivotal studies that demonstrated the efficacy of each treatment. If the results of a Japanese study were to be published, priority would be given to the Japanese study over a global study. In cases where some of the relevant values were not reached, real world data or hypothetical values were adopted. The details are presented in Table S1. The utilities are primarily referred to as systematic reviews. The systematic review used the MEDLINE, Embase, and Cochrane Library databases to systematically search the literature describing health state utility value (HSUV) in mNSCLC regardless of the treatment line (September 2016). It also cited the EQ-5D website and the School of Health and Related Research Utilities Database (SCHARRHUD) [25]. However, most data on the utility of each of the latest targeted treatments are unavailable. A complete list of model parameters and their sources is provided in Table S1.

### 2.5 Sensitivity analysis

One-way deterministic sensitivity analyses (DSAs) were performed to assess the effects of changing the model parameters. A range of values based on 95 % confidence intervals, or if not available, the utility values of probability and health varied by ±20 %, and the costs varied by ±30 % [16] (adjusted to the extent that there was no error in TreeAge Pro Healthcare). The distribution parameters are listed in Table S1. For the cost of F1CDx, the sum of the actual testing fee, evaluation provision fee, and range of the testing fee were adopted.

Probabilistic sensitivity analyses (PSAs) were performed using 1000 iterations of Monte Carlo simulations to examine the influence of parameter uncertainty on cost-effectiveness. The probabilistic distributions of the parameters used in the probabilistic sensitivity analysis are listed in Table S1.

## 3 Results

### 3.1 Base case results

The expected costs of Strategy1, Strategy2, and Strategy3 were USD 191,115, USD 191,806, and USD 195,198; JPY 28,956,821; JPY 29,061,492; and JPY 29,575,492, respectively (Table 1, USD 1 = JPY 151.39 as of April 1st, 2024). The expected LYs are 3.46, 3.54, and 3.54, respectively.

**Table 1:** Base case results for the cost-effectiveness analyses (LYs) and cost-utility analyses (QALYs).

Analysis	Comparator	Mean LY or QALY per patient	Mean costs per patient USD or JPY^b^	Incremental Cost (IC) USD or JPY^b^	Incremental Effectiveness(IE) LY or QALY	ICER(IC/IE) USD or JPY^b^/LY or QALY
**CEA**	**Strategy1**	3.46 LY	$191,115			
	With F1CDx after SOC		¥28,956,821			
	**Strategy2**	3.54 LY	$191,806	$691	0.08 LY	$8600/LY
	Without F1CDx		¥29,061,492	¥104,671		¥1,302,987/LY
	**Strategy3**	3.54 LY	$195,198			Dominated^a^
	With F1CDx at initial diagnosis		¥29,575,492			
**CUA**	**Strategy1**	2.56 QALY	$191,115			
	With F1CDx after SOC		¥28,956,820			
	**Strategy2**	2.61 QALY	$191,806	$691	0.05 QALY	$13,734/QALY
	Without F1CDx		¥29,061,492	¥104,671		¥,080,923/QALY
	**Strategy3**	2.61 QALY	$195,198			Dominated^a^
	With F1CDx at initial diagnosis		¥29,575,492			

United States Dollar (USD); Japanese Yen (JPY); cost-effectiveness analysis (CEA); cost-utility analysis (CUA); incremental cost-effectiveness ratio (ICER); life-years (LYs); quality-adjusted life-years (QALYs); incremental cost (IC); incremental Effectiveness (IE); standard of care (SOC).

a Dominated strategies are less effective than cheaper alternatives.

b 1 USD = JPY 151.39 as of April 1st, 2024.

QALYs were 2.56, 2.61, and 2.61, respectively. For both LY and QALYs, Strategy3 was dominated; therefore, it was excluded from subsequent analysis. For both CUA and CEA, Strategy3 and Strategy2 (single tests performed at initial diagnosis) resulted in the same gain in QALYs and LYs. This seems reasonable, because the treatment choice based on the test results did not change, regardless of whether it was a single test or F1CDx.

**Fig. 3:**
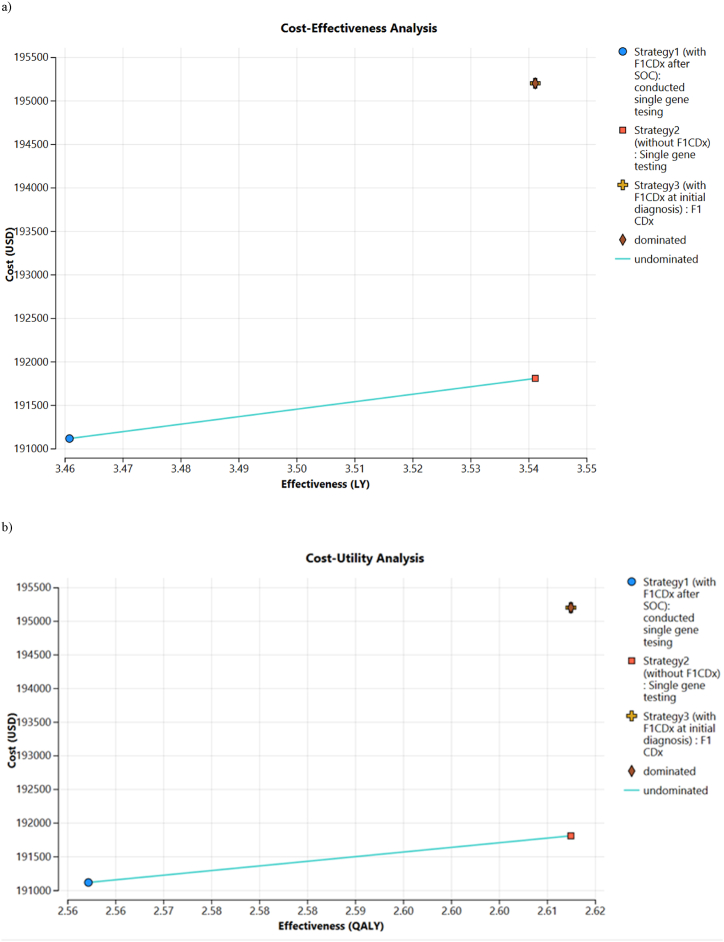
a) Cost-effectiveness analysis, b) Cost-utility analysis United States Dollar (USD), cost-effectiveness analysis (CEA), cost-utility analysis (CUA), life years (LYs), quality-adjusted life years (QALYs), and the standard of care (SOC).

Strategy2, which performed single-gene tests at initial diagnosis, had an incremental cost of USD 691 (JPY 104,671) and incremental effectiveness of 0.08 LY and 0.05 QALYs compared to Strategy1, which conducted F1CDx after standard treatment, with an ICER per LY of USD 8, 600 (JPY 1,302,987) and an ICER per QALY of USD 13,734 (JPY 2,080,934) (Table 1, Fig. 3(a and b)).

For cancer treatment, the ICER per QALY was lower than that of the WTP in Japan, which was estimated to be USD 45,900 (JPY 7,500,000) [26,27]. Given that Strategy3 was dominated, the introduction of F1CDx was not cost-effective in June 2019, either after standard treatment or at initial diagnosis, compared with single tests conducted simultaneously. The mean treatment cost per patient when multiple single tests were conducted at initial diagnosis was approximately JPY 100,000 lower than that when F1CDx was conducted after standard treatment.

### 3.2 Sensitivity analysis

**Fig. 4:**
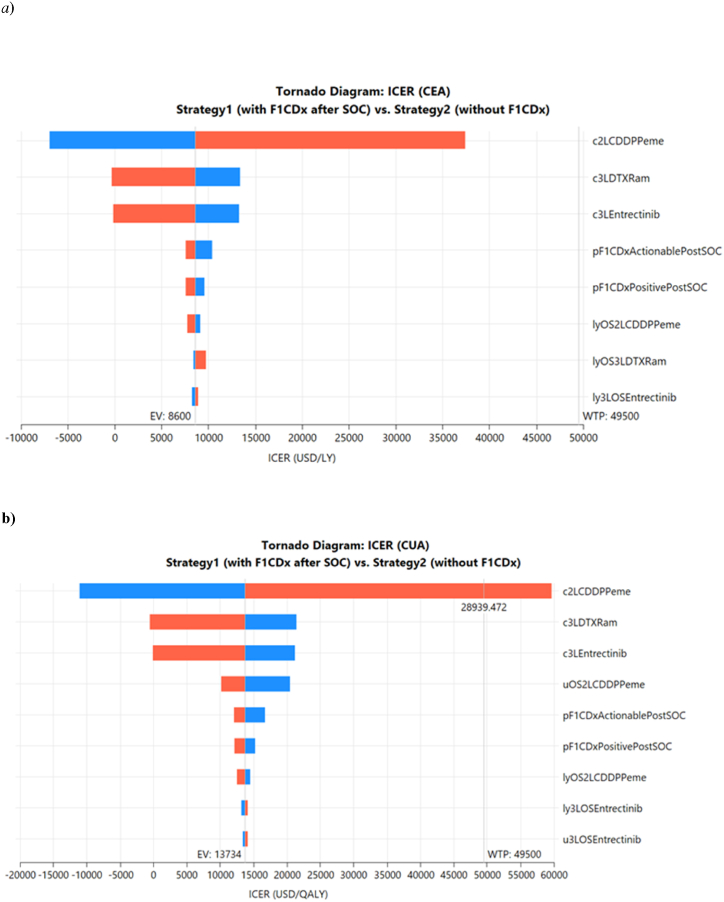
a) Deterministic sensitivity analysis for CEA (Strategy1 versus Strategy2). b) Deterministic sensitivity analysis for CUA (Strategy1 versus Strategy2) Blue (High) and Red (Low) are confidence intervals United States dollars (USD); cost-effectiveness analysis (CEA); cost-utility analysis (CUA); incremental cost-effectiveness ratio (ICER); willingness to pay (WTP); carboplatin (CBDCA); cisplatin (CDDP); docetaxel (DTX); ramucirumab (Ram); progression-free survival (PFS); overall survival (OS); standard of care (SOC) c: cost (USD/year), p: probability, u: utility, 1 L: 1st line, 2 L: 2nd line, 3 L: 3rd line.

**Fig. 5:**
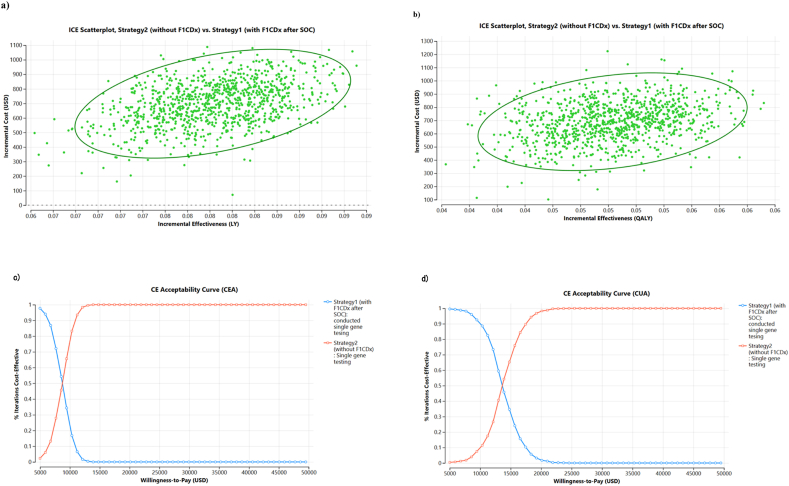
Probabilistic sensitivity analysis (Strategy2 with multiple-single tests at initial diagnosis vs Strategy1 with F1CDx after SOC) a) Scatter plot for CEA (LYs), b) Scatter plot for CUA(QALYs), c) Cost-effectiveness acceptability curve for CEA (LYs), and d) Cost-effectiveness acceptability curve for CUA(QALYs) United States Dollar (USD); cost-effectiveness analysis (CEA); cost-utility analysis (CUA); life-years (LYs); quality-adjusted life-years (QALYs); incremental cost-effectiveness ratio (ICER); standard of care (SOC); c: cost (USD/year).

Since Strategy3 was dominated by CEA and CUA, DSAs were performed for Strategy1 and Strategy2 (Fig. 4(a and b)). The results of the DSAs of Strategy1 against Strategy2 for CEA showed that the major uncertainty factors were, in descending order of impact, cost of second- and third-line treatments, probability that the F1CDx result was actionable, probability that the F1CDx result was positive for some genetic alterations, and survival years for second- and third-line treatments. The results of the DSA of Strategy1 against Strategy2 for CUA showed that the major uncertainty factors were, in descending order of impact, the cost of second- and third-line treatments, utility of second-line OS, probability that the F1CDx result was actionable, probability that the F1CDx result was positive for some genetic alterations, survival years for second- and third-line treatments, and utility of third-line OS. PSAs were conducted using these highly influential uncertainty factors in the DSA. The PSA results supported the results at each point estimate presented in the one-way sensitivity analysis (Fig. 5(a–d)).

## 4 Discussion

### 4.1 F1CDx conducted at initial diagnosis

As of June 2019, the ICER per QALY for performing multiple single tests at the initial diagnosis versus performing F1CDx after standard treatment was USD 13,734 (JPY 2,080,934). The strategy of performing F1CDx at the initial diagnosis was dominated by the other two strategies. If the WTP for anticancer treatment in Japan was USD 49,500 (JPY 7,500,000) per QALY for both diagnosis and treatment [27,28], the most cost-effective strategy in mNSCLC treatment at that time was to conduct multiple simultaneous single tests at the initial diagnosis, and F1CDx at the initial diagnosis was not an option from a health economic perspective. In addition, the QALYs gained from F1CDx at the initial diagnosis were the same as those obtained from multiple single tests at the initial diagnosis, and no patient benefit was derived from this strategy. The reason for setting the WTP for anticancer treatment in Japan at USD 49,500 (JPY 7,500,000)/QALY is that this is the standard amount for price reduction within the ICER when considering the WTP [27,28]. However, there are few references, and further discussion is required.

In a previous study, the cost-effectiveness of F1CDx for single-gene tests at initial diagnosis in the United States was moderate (USD 147,000/LY), which was considered acceptable because the WTP in the United States ranged from USD 50,000 to USD 200,000 per LY. Because our study did not include the cost of hospitalization or care for side effects, our results cannot be compared with those reported in the United States. However, in Japan, the cost-effectiveness of F1CDx implementation versus multiple single tests at the initial diagnosis is low and negative in terms of the clinical introduction of F1CDx.

### 4.2 F1CDx conducted after standard treatments

In Australia, the cost-effectiveness of ODxTT after standard treatment for mNSCLC compared to no ODxTT or BSC has been evaluated [16]. When ODxTT was conducted and targeted treatments were available, compared to BSC without ODxTT, the ICER per LY was AUD 485,199 (JPY 48,034,701; AUD1 = JPY 99 as of April 2024), which was not cost-effective considering the Australian WTP threshold per LY at that time, AUD 100,000. In an Australian study, the ICER per LY was considered high because the costs of hospital stay and side effect management were included. In the CUA, the ICER per QALY was AUD 489,338 (JPY 48,444,462), whereas in our study, the ICER per QALY or per LY for F1CDx after standard treatment was inferior to that for single tests at the initial diagnosis. Despite the differences between ODxTT and F1CDx, our study supports the previous prediction that NGS is not cost-effective after standard treatment. In the CUA, the uncertainty factor for Strategy1 to outperform Strategy2 was that the cost of second-line CDDP/Peme would be more than USD 28,939 based on the DSA results (Fig. 4b); however, since CDDP and Peme have been on the market for approximately 20 years, there is no such possibility in reality. No other factors were suggested to have the potential to exceed the WTP according to the results of the DSAs. The next most influential third-line treatments are targeted treatments and immunotherapies, which are likely to be launched in the future. Therefore, if the model focuses on the timing of F1CDx implementation, as in this case, the price of new treatments after the third-line treatment may become an issue in the future.

### 4.3 New findings

When targeted treatments were first introduced in clinical practice, the scheme was simple; a single-gene test was followed by the corresponding treatment. However, advances in diagnostic technology have made this scheme more complex, as NGS is performed, and targeted treatments are tailored to the results as appropriate. This has made it necessary to consider not only the clinical efficacy of each diagnosis and treatment but also health care economics. In our study, the results of HTA examining the timing of the clinical introduction of NGS, particularly F1CDx, for mNSCLC patients in Japan revealed, for the first time, all expected costs, LYs, and QALYs for each of the three diagnostic and treatment strategies from initial diagnosis to after standard therapy. In other words, we quantitatively evaluated which tests should be performed and when, in terms of both scientific effectiveness and healthcare economics.

The results of our study indicate that from the standpoint of patient benefit and healthcare economics, conducting multiple simultaneous single tests at initial diagnosis is preferable to conducting F1CDx after standard treatment or at initial diagnosis, as of June 2019. It was also found that introducing F1CDx after standard treatment was more realistic than introducing F1CDx at initial diagnosis, although it was not as cost-effective as multiple simultaneous single tests at initial diagnosis. Therefore, the decision of the Japanese government at the time of the clinical introduction of F1CDx to administer F1CDx after standard treatment, rather than at initial diagnosis, was reasonable from the perspective of maintaining the Japanese healthcare economy in the short term [28].

### 4.4 Policy implications

As noted in previous studies [17,18], the cost of treatment, rather than the cost of NGS, was the factor with the greatest impact on the ICER. This suggests that to optimize options in terms of both effectiveness and cost, measures to reduce the cost of treatment should be prioritized over the cost and timing of testing. It has also been suggested that if more actionable treatment options with F1CDx become available, the usefulness of F1CDx will increase, even after standard treatment. In future, CEA should be conducted using a comprehensive decision tree covering the period from initial diagnosis to post-standard treatment, assuming new options with lower treatment costs.

Since the introduction of F1CDx in clinical practice in June 2019, restrictions on insurance points and the timing of test implementation have been relaxed. It was announced that the insurance points for CGP tests would be changed to 44,000 points (JPY 440,000; 1 point = 10 JPY) from April 2022, and restrictions on the timing of calculations, such as when the specimens were submitted and when the results were explained, were eliminated [29]. Because of this change, if the patient's general condition worsened without treatment before the results of the expert panel became available, a situation in which JPY 480,000 was not paid could have been avoided. However, as of April 2024, it has not been disclosed whether the evaluation of this new policy option has been conducted using the HTA methodology. Thus, HTA should be applied to such situations, and the policy change should be evaluated quantitatively.

### 4.5 Limitations

This study has the following limitations; however, they are not expected to affect the ranking of each strategy. To keep the model as simple as possible, we counted up to third-line treatment in the case of F1CDx implementation after standard treatment, and up to secondary treatment in all other cases. This study limited the target genes in the CDx function of F1CDx to *EGFR* and *ALK*, which were approved as of June 2019, and did not evaluate the value of any increase in target genes beyond that date. Strictly speaking, the total treatment periods were not aligned. In addition, because we were unable to use a claims database such as the JMDC for treatment costs, we did not consider the cost of drugs and hospitalization to deal with side effects, nor did we consider the actual duration of treatment. Turnaround time (TAT) delays and additional clinical times were not considered. Therefore, the mean cost per patient and ICER may be lower than in reality. This study used PFS and OS as efficacy parameters; however, because OS is a parameter that includes the post-treatment effect and does not accurately reflect efficacy if the relevant treatment is discontinued, survival years and QALYs may be estimated to be longer than in reality. As there are no published figures specific to each targeted treatment and the scores for conventional chemotherapy are used, it is likely that the estimates are lower than they are, and the CUA for the portion using targeted treatments may be estimated to be lower. The reason for setting all cases to undergo re-biopsy before F1CDx implementation after standard treatment in this study was to simplify the complex decision tree, which was divided into three strategies, by minimizing branching based on the current situation where re-biopsy is realistic in Japan [30], In practice However, there may be cases in which re-biopsy cannot be performed because of the location of the lesion.

## 5 Conclusion

The results of this study revealed that from both patient benefit and health economic perspectives, in June 2019, multiple simultaneous single tests at initial diagnosis were preferable to F1CDx, either after standard treatment or at initial diagnosis. It was also found that introducing F1CDx after standard treatment was more realistic than introducing F1CDx at the time of initial diagnosis, although it was not as cost-effective as multiple simultaneous single tests at the time of initial diagnosis. Thus, the policy of introducing the CGP in Japan was appropriate from the perspective of preserving health economics in the short term. In addition, when aiming for a sustainable healthcare economy in Japan, the priority should be to consider measures to control treatment costs, rather than test costs and timing.

## Ethics declarations

Informed consent was not required for this study because we did not have access to patient data.

## Funding statement

This study did not receive any specific grants from funding agencies in the public, commercial, or nonprofit sectors.

## Data availability statement

There is no additional data available for this study.

## CRediT authorship contribution statement

**Naoko Shiraiwa:** Writing – original draft, Visualization, Project administration, Methodology, Formal analysis, Conceptualization. **Shingo Kano:** Writing – review & editing, Supervision, Conceptualization.

## Declaration of competing interest

The authors declare the following financial interests/personal relationships which may be considered as potential competing interests: Naoko Shiraiwa reports a relationship with Pfizer Japan Inc. that includes: employment and equity or stocks but has no interest in this research that would affect the work reported in this paper. Shingo Kano has no financial interests/personal relationships that could have influenced the work reported in this paper.
