# Supplementary material for: Cost-effectiveness of multigene sequencing test and treatment for metastatic non-small cell lung cancer: A unique setting in the initial adoption phase in Japan allowing testing only after standard treatment
Source: Heliyon. 2024 Sep 19;10(19):e37867. doi: 10.1016/j.heliyon.2024.e37867 (PMC11471211; doi:10.1016/j.heliyon.2024.e37867)
Supplement: Multimedia component 1 [file mmc1.docx]

**Table S1 Model inputs**

| **Model variable** | **Value** | **Range** | **Distribution** | **Reference or Remarks** |
| --- | --- | --- | --- | --- |
| c1LAlectinib | 64028 | ±30% | Gamma | Kyoto Encyclopedia of Genes and Genomes (KEGG) medicus database (<https://www.kegg.jp/kegg/medicus.html>) at Jun 2019, [31] |
| c1LCBDCA_Peme_Pembro | 115292 | ±30% | Gamma | same as above, [32–34] |
| c1LOsimertinib | 49228 | ±30% | Gamma | same as above, [35] |
| c2LCDDP_Peme | 14388 | ±30% | Gamma | same as above, [33,36] |
| c2LLorlatinib | 62826 | ±30% | Gamma | same as above, [37] |
| c3LDTXRam | 54855 | ±30% | Gamma | same as above, [38,39] |
| c3LCDDP_Peme | 14388 | ±30% |  |  |
| c3LEntrectinib | 71800 | ±30% | Gamma | same as above, [40] |
| cALKTesting | 165 | ±30% | Gamma | the medical fee database (<http://shinryohoshu.mhlw.go.jp/shinryohoshu/>) at Jun 2019 |
| cBiopsy | 317 | ±30% | Gamma | same as above |
| cEGFRTesting | 139 | ±30% | Gamma | same as above |
| cF1CDx | 3696 | ±30% | Gamma | same as above |
| ly3LOSEntrectinib | 1.75 | 1.24-NE | Pert | [41], estimated median OS |
| ly3LPFSEntrectinib | 0.92 | 0.67-NE | Pert | [41] |
| lyOS1LAlectinib | 5.88 | 5.88-NE | Pert | [42][43,44], relevant values were not reached. So, 95% CI lower limit of [36] was adopted. |
| lyOS1LOsimertinib | 3.217 | 2.875-3.483 | Pert | [45] |
| lyOS2LCDDPPeme | 0.858 | 0.817-0.933 | Pert | [46] |
| lyOS2LLorlatinib | 2.74 | 1.56-NE | Pert | Not reached in[47], so adopted[48] |
| lyOS3LDTXRam | 1.26 | 1.04-2.21 | Pert | [49] |
| lyPFS1LAlectinib | 2.842 | 1.842-NE | Pert | [50] |
| lyPFS1LCBDCAPemePembro | 0.733 | 0.633-0.767 | Pert | [51] |
| lyPFS1LOsimertinib | 1.575 | 1.267-1.783 | Pert | [52] |
| lyPFS2LCDDPPeme | 0.40 | 0.38-0.44 | Pert | [46] |
| lyPFS2LLorlatinib | 0.458 | 0.225-0.750 | Pert | [47] |
| lyPFS3LDTXRam | 0.435 | 0.293-0.581 | Pert | [49] |
| pALKPositive | 0.05 | ±20% | Beta | [1] |
| pEGFRPositive | 0.5 | ±20% | Beta | [1] |
| pF1CDxActionablePostSOC | 0.133 | ±20% | Beta | [6] |
| pF1CDxPositivePostSOC | 0.594 | ±20% | Beta | [6] |
| Prob1LPFSAlectinibToDead | Probtoprob(0.5;1/48) | ±20% | Beta | Parameters within TreeAgeProHealthcare |
| Prob1LPFSCBDCAPemePembroToDead | Probtoprob(0.5;1/33) | ±20% | Beta | same as above |
| Prob1LPFSOsimertinibToDead | Probtoprob(0.5;1/27) | ±20% | Beta | same as above |
| Prob2LPFSCDDPPemeToDead | Probtoprob(0.5;1/30) | ±20% | Beta | same as above |
| Prob2LPFSLorlatinibemeToDead | Probtoprob(0.5;1/14) | ±20% | Beta | same as above |
| Prob3LPFSEntrectinibToDead | Probtoprob(0.5;1/48) | ±20% | Beta | same as above |
| pTrans1LPFSAlectinibProgressed | Disttransprob("Dist_1LPFSAlectinib";_stage/CyclesPerYear;1/CyclesPerYear) | ±20% | Beta | same as above |
| pTrans1LPFSCBDCATo2LPFSCDDPPeme | Disttransprob("Dist_1LPFSCBDCAPemePembro";_stage/CyclesPerYear;1/CyclesPerYear) | ±20% | Beta | same as above |
| pTrans1LPFSOsimertinibProgressed | Disttransprob("Dist_1LPFS_Osimertinib";_stage/CyclesPerYear;1/CyclesPerYear) | ±20% | Beta | same as above |
| pTrans2LOSCDDPPemeToDead | Disttransprob("Dist_2LOSCDDPPeme";_tunnel/CyclesPerYear;1/CyclesPerYear) | ±20% | Beta | same as above |
| pTrans2LOSLorlatinibToDead | Disttransprob("Dist_2LOSLorlatinib";_tunnel/CyclesPerYear;1/CyclesPerYear) | ±20% | Beta | same as above |
| pTrans2LPFSCDDPPemeToProgressed | Disttransprob("Dist_2LPFSCDDPPeme";_tunnel/CyclesPerYear;1/CyclesPerYear) | ±20% | Beta | same as above |
| pTrans2LPFSLorlatinibToProgressed | Disttransprob("Dist_2LPFSLorlatinib";_tunnel/CyclesPerYear;1/CyclesPerYear) | ±20% | Beta | same as above |
| pTrans3LOSDTXRamToDead | Disttransprob("Dist_3LOSDTXRam";_tunnel/CyclesPerYear;1/CyclesPerYear) | ±20% | Beta | same as above |
| pTrans3LOSEntrectinib | Disttransprob("Dist_3LOSEntrectinib";_tunnel/CyclesPerYear;1/CyclesPerYear) | ±20% | Beta | same as above |
| pTrans3LPFSDTXRamToDead | Probtoprob(0.5;1/18) | ±20% | Beta | same as above |
| u3LOSEntrectinib | 0.55 | ±20% | Beta | [25] |
| u3LPFSEntrectinib | 0.81 | ±20% | Beta | [25] |
| uOS1LAlectinib | 0.321 | ±20% | Beta | [25] |
| uOS1LCBDCAPemePembro | 0.67 | ±20% | Beta | [53] |
| uOS1LOsimertinib | 0.74 | ±20% | Beta | [25] |
| uOS2LCDDPPeme | 0.59 | ±20% | Beta | [53] |
| uOS2LLorlatinib | 0.59 | ±20% | Beta | [53] |
| uOSDTXRam | 0.42 | ±20% | Beta | [53] |
| uPFS1LAlectinib | 0.804 | ±20% | Beta | [53] |
| uPFS1LCBDCAPemePembro | 0.71 | ±20% | Beta | [53] |
| uPFS1LOsimertinib | 0.83 | ±20% | Beta | [25] |
| uPFS2LCDDPPeme | 0.74 | ±20% | Beta | [53] |
| uPFS2LLorlatinib | 0.82 | ±20% | Beta | [54] for ALK-TKI as 2L |
| uPFSDTXRam | 0.61 | ±20% | Beta | [25] |

United States dollars (USD); c: cost (USD/year); ly: life-year (Y); p: probability; u: utility; NE: not estimated; NR: not reached; 1L: 1^st^ line, 2L: 2^nd^ line, 3L: 3^rd^ line, Dist: distribution; Prob: probability; trans: transition

Carboplatin (CBDCA), cisplatin (CDDP), docetaxel (DTX), progression-free survival (PFS), overall survival (OS), standard care (SOC)

Pemetrexed (Peme); Pembrolizumab (Pembro)

The life-year range was adjusted based on 95% CI for each efficacy data point. Because annual treatment costs were required for analysis by TreeAge Pro Healthcare, treatment costs were calculated for one year of continued administration at the recommended dosage for each treatment. To calculate body surface area, the patient’s height and weight were assumed to be 170 cm and 65 kg, respectively. With respect to each parameter, except treatment costs, the most recent version was used. As of June 2019, when F1CDx was launched, it was used to cover treatment costs.
